# Prevention of radiochemotherapy-induced toxicity with amifostine in patients with malignant orbital tumors involving the lacrimal gland: a pilot study

**DOI:** 10.1186/1748-717X-3-22

**Published:** 2008-09-01

**Authors:** David Goldblum, Pirus Ghadjar, Juergen Curschmann, Richard Greiner, Daniel Aebersold

**Affiliations:** 1 Department of Ophthalmology, University Basel, University Hospital Basel, Switzerland; 2 Department of Radiation Oncology, University Bern, Inselspital, Bern, Switzerland

## Abstract

**Background:**

To use amifostine concurrently with radiochemotherapy (CT-RT) or radiotherapy (RT) alone in order to prevent dry eye syndrome in patients with malignancies located in the fronto-orbital region.

**Methods:**

Five patients (2 males, 3 females) with diagnosed malignancies (Non-Hodgkin B-cell Lymphoma, neuroendocrine carcinoma) involving the lacrimal gland, in which either combined CT-RT or local RT were indicated, were prophylactically treated with amifostine (500 mg sc). Single RT fraction dose, total dose and treatment duration were individually adjusted to the patient's need. Acute and late adverse effects were recorded using the RTOG score. Subjective and objective dry eye assessment was performed for the post-treatment control of lacrimal gland function.

**Results:**

All patients have completed CT-RT or RT as indicated. The median total duration of RT was 29 days (range, 23 – 39 days) and the median total RT dose was 40 Gy (range, 36 – 60 Gy). Median lacrimal gland exposure was 35.9 Gy (range, 16.8 – 42.6 Gy). Very good partial or complete tumor remission was achieved in all patients. The treatment was well tolerated without major toxic reactions. Post-treatment control did not reveal in any patient either subjective or objective signs of a dry eye syndrome.

**Conclusion:**

The addition of amifostine to RT/CT-RT of patients with tumors localized in orbital region was found to be associated with absence of dry eye syndrome.

## Background

Xerostomia, mucositis and dysphagia are known serious adverse reactions associated with radiotherapy (RT) or radiochemotherapy (CT-RT) of tumors localized in the head and neck region. Of particular concern is xerostomia, which develops acutely, but persists chronically afterwards and may lead to serious complications. Another serious toxic reaction to RT is xerophthalmia (dry eye syndrome/keratoconjunctivitis sicca), which can develop after local irradiation of the fronto-orbital region and can lead to severe visual impairment. Generally, the burden caused by RT-induced toxic reactions is not only detrimental for the patient's quality of life and the compliance with treatment, but may also have considerable pharmaco-economic consequences because of the costs incurred by loss of productivity, hospitalization and, frequently, expensive supportive measures [1]. It is therefore a major and desirable goal of the RT to achieve tumor remission without compromising the general well-being of the patient.

Amifostine is a unique drug selectively protecting non-affected tissues from CT-RT induced toxicity. Its efficacy in preventing toxic damages induced by irradiation of tumors localized in the head and neck region, particularly in the naso-oropharynx, has been demonstrated in several clinical trials [2-7].

We report here our experience with amifostine, which was used as a prophylactic measure during RT of malignant tumors localized in the fronto-orbital region, involving the ipsilateral lacrimal gland.

## Methods

### Patient sample

Five Patients, who were admitted to the department of radiation oncology, Inselspital Bern with histopathologically characterized unilateral malignant tumors of the orbital region and who gave informed consent for treatment with RT or CT-RT combined with amifostine, where included in this study. None of the patients had a history of dry eye, sarcoidosis, or thyroid associated eye disease.

### Treatment

According to the diagnosis and histopathological findings for each patient a 3D RT planning was performed. Using dose volume histograms (DVH) minimum and maximum dose exposure of the lacrimal gland was determined. Two patients received chemotherapy according to the standard CHOP-protocol. In one patient, chemotherapy preceded RT and in another it was given concurrently with RT.

Amifostine in a dose of 500 mg was given subcutaneously 30 min prior to each RT-fraction to all patients

### Lacrimal gland assessment

In order to check for possible existence of dry eye syndrome in the post – treatment period lacrimal gland function was systematically assessed in all patients. The objective ophthalmic tests were performed at different time intervals after the termination of RT. The longest interval between RT and the tests was 88 weeks (pat. No 4) and the shortest 9 weeks (pat. No 5), median interval was 57.5 weeks.

The following objective tests were performed: a) Schirmer I (with and without local anesthesia), which measures basic and reflex-secretion of the lacrimal gland, b) Schirmer II (nasal stimulation) test, which measures stimulated secretion of the lacrimal gland c) Tear-film break up time (BUT), which measures the time of onset of a random appearance of the first black spots on the cornea after one single palpebral (eyelid-blinking) closure under standardized conditions, d) corneal and conjunctival fluorescein staining and e) Rose bengal staining of the conjunctiva.

A single examiner performed all of the tests and interviews during the examination in the department of ophthalmology. In all tests of the non-irradiated, contralateral eye served as control except in patient 1. The procedures are briefly described below.

#### Dry eye symptom questionnaire

Patients were first interviewed to survey the frequency of occurrence of various dry eye symptoms including dryness, grittiness, redness, excess tearing or watery eyes, sensitivity (to smoke, wind, air conditioning) and soreness. Response categories used for analyses included never, seldom (two to three times per week), often (four to five times per week), and always (everyday).

#### Schirmer I test

The Schirmer I test (Laboratoires H. Faure, Annonay, France) was performed without anesthetic (5 min, open eye) and the strip was placed over the inferior lid margin towards the lateral canthus. Abnormal values were defined as < 5 mm in 5 min for the Schirmer test. Baseline secretion is determined essentially as described above, except that the eye is anesthetized 2 minutes before the exam with one drop of local anesthetic (Novocaine 2%, Inselspital, Bern).

#### Schirmer II test

The procedure is similar to Schirmer I test, except that after the suspension of the paper strip in the inferior fornix of a non-anaesthetized eye, the nasal mucosa is stimulated for 2 minutes with a cotton tip frotting on the nasal mucosa between the concha medalis and the concha inferior [8].

#### Tear-film break up time (BUT)

The tear-film break-up time measurement was taken using the cobalt blue illumination on the slit-lamp and a 3 mm wide scanning beam in each eye. Fluorescein sodium was instilled on the inferior palpebral conjunctiva using a Fluorescein Ophthalmic Strip (Haag-Streit, Köniz, Switzerland). The latency to the first, random appearance of a black spot, in the otherwise yellow-green colored surface of the eye after complete closure of the eye-lid, is measured. The normal latency to the first black-spot appearance is 15 – 35 sec and the values below 10 indicate an insufficient lubrification of the corneal surface.

#### Corneal and conjunctival fluorescein staining

Fluorescein sodium was instilled on the inferior palpebral conjunctiva using a Fluorescein Ophthalmic Strip (Haag-Streit, Köniz, Switzerland). Following instillation of fluorescein, the patient was instructed to blink several times. The slit-lamp cobalt blue was used in the assessment of tear break-up time and fluorescein staining to enhance the appearance of the fluorescein. Staining was recorded for the cornea and the conjunctiva. The cornea and adjacent exposed bulbar conjunctiva was graded using a 0–3 scale resulting in a total staining score. Abnormal staining (fluorescein and rose bengal) was classified as greater than or equal to a score of 3 [9].

#### Rose bengal staining of the conjunctiva

Rose bengal staining of the conjunctiva was performed by using a Rosets TM Rose Bengal Ophthalmic Strip (Chauvin Pharmaceuticals Ltd, Brentwood, UK) wetted with non-preserved buffered saline and instilled on the inferior bulbar conjunctiva. Grading for rose bengal staining was described previously [9]. A score 3 or higher was considered pathological [10].

### Adverse events

Acute and late adverse effects were recorded using the Radiation Therapy Oncology Group (RTOG) score . Spontaneously reported signs of eye discomfort such as for instance tears, redness or dryness of the eye, were regularly recorded. It is to note, that there were no subjective or objective signs of eye discomfort in any patient prior to CT-RT or RT.

## Results

### Patients characteristics

Demographic characteristics of patients are presented in Table 1. Incisional histopathology confirmed malignant tumors of different tumor entities (Non-Hodgkin B-Cell Lymphoma, n = 4; neuroendocrine carcinoma, n = 1) with expansion into the orbita. Two patients were male, three were female, median age was 63 years (range, 28 – 79 years).

**Table 1:** Characteristics of the patient sample

	Clinical factors			
Pat No	Gender	Age	Diagnosis	Tumor localization

1	Male	28	Neuroendocrine carcinoma	Frontobasal part of the right nasal sinus
2	Female	60	Non-Hodgkin B-cell Lymphoma, low grade (St. IAE)	Right lacrimal gland
3	Female	69	Non-Hodgkin B-cell Lymphoma, low grade, (St. IAE; Malt-Type)	Right orbita
4	Female	63	Non-Hodgkin-B-cell Lymphoma, high grade, (St. IAE)	Right M. temporalis, with orbital expansion
5	Male	79	Non-Hodgkin B-cell Lymphoma, low grade, (St. IAE)	Right palpebra

Abbreviations: St. = stage; IAE = staging according to the Ann-Arbor-classification; M. = musculus

### Clinical outcome

Table 2 illustrates the details of the treatment modalities and the outcome of treatment in each patient. Combined CT-RT was given in two and RT alone to 3 patients. The median RT treatment period was 29 days (range, 23 – 39 days). The median total RT dose applied to the reference point according to the International Comission on Radiation Units and Measurements (ICRU) 10 was 40 Gy (range, 36 – 60 Gy). Median lacrimal gland exposure on the treated side was 35.9 Gy (range, 16.8–42.6 Gy). All patients completed the treatments according to the planned protocol. In all patients the response to therapy was excellent with complete response in 2 patients and a partial remission in the remaining three patients.

**Table 2:** Treatment modalities and outcome

	Treatment factors				
Pat No	Radio chemotherapy CT/RT*	RT (total duration days)	Total RT Dose† Gy	Median RT dose Lacrymal gland Gy right/left	Clinical Outcome

1	6/39		60	16.8/16.5	partial remission
2		35	36	35.9/0	complete remission
3		29	40	42.6/5.4	complete remission
4	3/34		46	35.4/0	partial remission
5		23	36	36.3/0.54	partial remission

*CT = chemotherapy; RT = radiotherapy; CT/RT = total cycles/total duration days; † for the RT reference point

A brief description of the histories is given below.

#### Patient No 1

Presented himself with progressive protrusion of his left eye, anosmia and difficulties with nasal breathing. CT and MRI examinations showed a frontobasal tumorous formation with extension from frontal sinus to sphenoidal sinus with protrusion to orbital and nasal cavity. There were also large osseous destructions of the lamina cribrosa and the medial orbital and maxillar sinus walls. Histopathological findings confirmed the diagnosis of a malignant neuroendocrine small cell carcinoma in advanced stage.

The patient was hospitalized and sequential CT-RT initiated. He was treated according to the CHOP-protocol with a good and rapid response after 1 cycle. Radical, frontobasal radiotherapy with a total dose of 60 Gy, applied simultaneously with the first and second out of totally six cycles of chemotherapy with Cisplatin 60 mg iv and Etopophos 240 mg iv led to almost complete regression of the tumorous tissue.

In the course of the RT the patient developed an acute grade II enoral mucositis, conjunctivitis and rhinitis, which regressed without further consequences after topical treatment.

#### Patient No 2

Underwent surgical intervention and subsequent external beam RT (total dose 50 Gy) of the Th-3-5 region because of metastatic, epidural tumors with compression of the spinal cord. 3 years later she presented herself again for consultation because of a massive palpebral edema, ptosis and exophthalmus of the right eye. Histopathological findings confirmed the diagnosis of a low-grade Non-Hodgkin B-Cell Lymphoma, stage IAE, with orbital expansion. A RT was performed with a total dose of 36 Gy. There were no toxic reactions found. One and a half year later the patient was in complete remission.

#### Patient No 3

Suffered from a progressive exophthalmus in her right eye for several years. The MRI showed an expansive tumorous mass in the right orbital cavity with intracranial spreading. Histopathologically findings confirmed the diagnosis of a Non-Hodgkin B-Cell Lymphoma, stage IAE, from the mucosa associated lymphatic tissue (MALT) type. Local radiotherapy of the right orbital region was performed to a total dose of 40 Gy. 27 weeks later the patient showed a complete remission of the tumor.

Radiotherapy was well tolerated. The only adverse reactions were grade I – skin reactions, a mild transitory conjunctivitis and a localized alopecia without consequences.

#### Patient No 4

Presented herself for a progressive edema in the right temporal region. A biopsy revealed a diffuse Non-Hodgkin B-Cell Lymphoma, stage IAE, with infiltration of the temporal muscle and expansion into the orbit. CHOP-protocol was initiated and completed in 3 cycles. Additionally, she received local external beam RT of the temporal region for consolidation with a total dose of 46 Gy

Until the end of the RT, except for a mild skin erythema, no other toxic reactions or complications were found. However, after 7 applications of amifostine (12 days after the start of RT) an allergic reaction to amifostine developed in the form of a generalized skin rash and edema of the face and neck. After stopping amifostine, the patient recovered without consequences. The post RT-control of the patient showed partial remission of the tumor.

#### Patient No 5

Presented himself with a progressive, persistent edema of the right eyelid, which had started 3 years ago. Histopathology confirmed the diagnosis of a low grade Non-Hodgkin B-Cell Lymphoma, stage IAE. Local RT of the right palpebral region was performed to a total dose 36 Gy.

There were no major toxicities during the RT treatment. Mild skin erythema of the right eyelid was the only adverse reaction. The post RT-control of the patient showed partial remission of the tumor.

### Adverse reactions

As seen from the case histories there were no major toxicities during the RT treatment and only few, mostly mild and transitory adverse events were recorded. One patient however showed, an allergic reaction to amifostine with generalized skin rash and edema of the face and neck which disappeared after halting amifostine.

### Post-treatment ophthalmologic control

The ophthalmic exams were performed at different time intervals after the termination of RT. The longest interval between finished RT and the exams was 88 weeks (pat. No 4) and the shortest 9 weeks (pat. No 5). The results of the post treatment-control of lacrimal gland function are illustrated in Table 3. As seen there were no clinically relevant impairments of glandular function, which would indicate serious dry-eye syndrome. Seldom feeling of a dryness of the exposed eye (n = 1), often tears (n = 1) and often eye-burning (n = 1) were the only subjective and reported complaints.

**Table 3:** Post-treatment lacrimal gland function

	Opthalmologic tests					
Pat No	Follow-up time (weeks after RT)	Schirmer I with anesthetic OD/OS	Schirmer I (without anesthetic) OD/OS	Schirmer II (nasal stimulation) OD/OS	BUT OD/OS	staining score

1	57.5	35/35	10/9	2/8	10/10	1 (OS)
2	77	5/10	5/7	7/11	15/15	1 (OD)
3	27	19/11	24/25	17/17	4/9	1 (OD)
4	87.7	5/18	1/7	9/17	15/15	2 (OD)
5	8.7	20/10	17/10	not performed	6/6	0

Abbreviations: RT = radiotherapy; OD = right eye; OS = left eye; BUT = break up time

## Discussion

Selective protective effects of amifostine against RT induced side effects localized in various body organs (lungs, rectum, cervix, ovaries) have been demonstrated in numerous clinical trials [6]. Its efficacy in patients with malignancies located in the head and neck region is, however, of particular importance. Ionizing radiation of this region impairs salivary and lacrimal gland functions, which has acute and long-term consequences for the patient. Proper lubrification of oral mucosa is a physiological prerequisite for normal chewing, swallowing, speaking, dental health and, last but not least, sleep [11,12]. Dryness of the eye, besides the discomfort, may lead to severe visual impairment due to the corneal damage. One study has reported the occurence of mild dry eye syndrome in 21% of patients after radiotherapy for stage IAE orbital lymphoma after a total dose of 30–51 Gy [13]. However in this study no dose volume histogram analysis for dose exposure of the lacrimal gland was performed and the data is thus not directly comparable to our study.

The experience with amifostine in patients with head and neck cancer is at present based on several studies. In a large (N = 315), randomized, comparative trial in patients with predominantly (≥75%) parotid gland carcinoma, Brizel et al. [5] have shown that by comparison to RT alone the concurrent amifostine-RT treatment reduced the overall incidence of grade ≥2 xerostomia from 78% to 51%. In another comparative study in 50 patients with mainly naso-oro-pharyngeal tumor localization, Antonadou et al. have shown considerable reduction of acute (mucositis and dysphagia) and late (xerostomia) RT-toxicities in the amifostine treated group (n = 22). In the control group 73.9% of patients (n = 23) developed grade 2 xerostomia, whereas in the amifostine-RT group there were only 27.2% patients with mild grade 2 xerostomia. Similar results were also reported in an earlier trial by Büntzel et al. In none of these studies amifostine treatment compromised the outcome of RT. In contrast, by comparison to the controls, the tumor remission rate in amifostine treated patients was, as a rule, higher (up to 90%) [14-16]. To the best of our knowledge, no clinical reports with amifostine in protecting lacrimal gland function from the irradiation of the orbital region have been reported.

In a rabbit model Beutel et al. evaluated the effect of amifostine in the tear gland and found morphological and functional evidence of its radioprotective properties [17].

Even though limited to five patients only, our observation is suggesting high success of combined amifostine-RT-treatment regarding tumor remission. Two out of five patients have achieved complete remission and the remaining three a partial remission of the tumors. Further, none of the patients experienced any major toxic reaction to RT. In particular, neither subjective complaints nor objective findings of the post-treatment ophthalmologic tests have revealed signs of a dry-eye syndrome.

As a matter of fact, we can not exclude the possibility that the lacrimal gland in our patients was exposed to a insufficiently high irradiation dose to induce any serious functional damage of the gland. Systematic investigations of the irradiation dose-effect relationship in cases of orbital tumors have not yet been done. In his review about the tolerance of the normal tissue to therapeutic radiation Emami [18] refers to the experiences related to the eye and parotid gland exposure to radiation. These observations seem to suggest a very steep dose-response curve at least for the eye. The quoted study of the exposure of the retina to 45–50 Gy led to detectable damages and that of 65 Gy and more to visual loss. For the parotid gland his estimate of the tolerance dose (TD) 5/5 (normal tissue complication probability at 5% within 5 years after radiotherapy), based on the reviewed studies was 32 Gy.

## Conclusion

Our results indicate that the addition of Amifostine to RT-/CH-RT in patients with tumors localized in orbital region is safe and associated with absence of dry eye syndrome. Our data encourage further studies of concurrent amifostine therapy.

This result is in accordance with the results of other clinical studies showing radioprotective effect of amifostine in patients with malignancies located in the head and neck region and radiation exposure involving the parotid gland.

## Competing interests

The authors have no personal financial or non-financial interests in any of the mentioned substances, companies or competing companies related to the study.

## Authors' contributions

DG, PG and JC performed the acquisition of data and helped to draft the manuscript. RG and DA made substantial contributions to conception and design of the study and helped to draft the manuscript. All authors have given final approval of the version to be published.
